# Chemical and biological investigations of *Limonium axillare* reveal mechanistic evidence for its antidiabetic activity

**DOI:** 10.1371/journal.pone.0255904

**Published:** 2021-08-06

**Authors:** Essam Abdel-Sattar, Manal M. Shams, Marwa M. Abd-Rabo, Nehad Mahmoud, Engy A. Mahrous

**Affiliations:** 1 Department of Pharmacognosy, Faculty of Pharmacy, Cairo University, Cairo, Egypt; 2 Department of Medicinal Plants and Natural Products, National Organization of Drug Control and Research, Giza, Egypt; 3 Department of Hormone Evaluation, National Organization of Drug Control and Research, Giza, Egypt; Bangabandhu Sheikh Mujibur Rahman Agricultural University, BANGLADESH

## Abstract

Root and bark of *Limonium axillare* (Forssk) Kuntze are used as antidiabetic remedies in parts of East Africa, but this activity has never been fully investigated. To validate its ethnobotanical use, we compared the chemical and pharmacological profiles of the ethanolic extracts of *L*. *axillare* root (REE) and aerial parts (AEE). Administration of REE (500 mg kg^-1^) reduced streptozotocin-induced hyperglycemia by 44%, restored serum insulin levels, reestablished Glut2 and Glut4 expression and ameliorated pancreatic tissue damage in diabetic rats. *In vitro* studies revealed a strong radical scavenging effect, α-glucosidase, and α-amylase inhibition activity of REE at IC_50_ at 25.2, 44.8 and 89.1μg/mL, respectively. HPLC analysis identified ten phenolic compounds in REE with umbelliferone as the major constituents at 10 ± 0.081 mg/g of extract. Additionally, six compounds were isolated from REE including, *β*-sitosterol-3-palmitate, *β*-sitosterol, myricetin and gallic acids with two new tetrahydrofuran monoterpenes; 2-isopropyl- 3,4,4, trimethyl-tetrahydrofuran (**3**), and 2-isopropyl-4-methyl-tetrahydrofuran-3,4 dicarboxylic acid (**4**), the latter was revealed by molecular docking to be a good ligand to glycerol-3-phosphate dehydrogenase a key enzyme in glycolysis.

## Introduction

Diabetes mellitus (DM) is an epidemic metabolic disease characterized by disruption of glycemic homeostasis leading to hyperglycemia, the hallmark of DM. Poorly controlled DM can lead to many complication including micro and macrovascular diseases, neuropathy, diabetic kidney disease and impairment of the immune system. At present, optimized glucose control is recognized as the best approach to reduce the risk of diabetes chronic complications [1, 2]. In areas where access to expensive antidiabetic medication is limited, medicinal plants play an important role in limiting the progress and complications of diseases such as DM [3, 4].

*Limonium axillare* (Forssk) Kuntze (Family Plumbaginaceae) is a small shrub distributed in desert and coastal land in East Africa especially in Egypt, Eritrea and Sudan [5] and throughout the Arabian Peninsula where it is known as "Qataf" and "Shelail" [6]. In Eastern Egypt, roots and barks of the plant are used as antidiabetic remedies [7], while in the Arab Peninsula, the decoction of the fresh plant is used to treat depression [8] and as astringent to treat diarrhea [6, 9]. Apart from its ethnobotanical use, previous investigations concluded that the methanolic extract of *L*. *axillare* has strong antioxidant activity [10]. Also its butanol extract displayed strong antibacterial and antifungal activity when compared to chloramphenicol and nalidixic acid, respectively [11]. The alcohol extract and two phenolic compounds obtained from *L*. *axillare* leaves have also shown good cytotoxic activity in both *in vitro* and *in vivo* models [12]. Leaves of *L*. *axillare* were found to contain several flavonoids, namely, apigenin, apiin, luteolin, quercetin, myricetin and kaempferol, in addition to phytosterols, namely, *β*-sitosterol, stigmasterol and *β*- sitosterol glucoside [13]. Meanwhile, the root bark is rich in phenolic acids including gallic, ferulic and isoferulic acid, and a number of coumarins including bergapten, umbelliferone and imperatorin [12].

Since no previous studies were reported to validate the ethnobotanical use of *L*. *axillare* as an anti-diabetic remedy, this study was designed to chemically investigate the ethanolic extracts obtained from the root system (REE) and the aerial part (AEE) and to correlate their chemical compositions to their antidiabetic activity through *in vivo*, *in vitro* and *in silico* studies. Six compounds were later isolated from the more active root extract resulting in the identification of two novel tetrahydrofuran derivatives.

## Material and methods

### Chemicals

Streptozotocin (STZ), acarbose, metformin (MF), *p*-nitophenylglucopyranoside, α- glucosidase (*Saccharomyces cerevisiae*), porcine pancreatic α-amylase were purchased from Sigma-Aldrich, St’ Louis, MO, USA. Folin-Ciocalteu reagent was purchased from Loba chemie, Mombai, India. External standards for HPLC analysis were either isolated from the plant or supplied by National Organization of Drug Control and Research (NODCAR), Egypt. All solvents were of analytical grade and purchased from Adwic company, Egypt. Acetonitrile and trifluoracetic acid were of HPLC grade and purchased from Sigma-Aldrich (MO, USA). Stationary phases for chromatographic separations were purchased from Merck (Darmstadt, Germany). NMR spectra were acquired using Brucker, Avance, 400 MHz. (MA, USA). MS data were recorded on Shimadzu LCMS-IT-TOF spectrometer (Kyoto, Japan).

### Plant material

Flowering aerial parts and roots of *Limonium axillare* (Forssk) Kuntze were collected from the Suez Canal road in January 2017 (29° 66’ 65’’ N, 32° 33’ 54’’ E). The plant was authenticated by Prof. Azza Mohamed Hosney El -Hadidy, Professor of Taxonomy and Flora, Department of Botany, Faculty of Science, Cairo University. A voucher specimen (Voucher no.: 8-3-2018) is deposited in Department of Pharmacognosy, Faculty of Pharmacy, Cairo University.

### Preparation of plant extracts

The air-dried powdered root and aerial parts (1.2 kg each) were extracted separately by repeated cold maceration with 70% ethanol (3 x 7 liters) to allow the extraction of phytoconstituents with different solubilities. The extracts were concentrated using vacuum to give 284 g and 154 g of residue of aerial part ethanolic extract (AEE) and root ethanolic extract (REE), respectively. The residue of the two extracts (50 g each) were suspended in water and subjected separately to successive liquid-liquid fractionation with *n*-hexane, chloroform, ethyl acetate (EtOAc) and *n*-butanol saturated with water to allow fractionation of plant constituents according to their different polarities. Different extracts were separately evaporated, weighted and stored at -4°C for further investigation.

### Determination of total phenolic and flavonoid contents

The total phenolic content (TPC) of the extracts was determined using the Folin–Ciocalteu method as described by Mruthunjaya and Hukkeri [14]. The absorbance was measured at 765 nm and TPC was expressed as mg equivalent of gallic acid per gram of extract (mg GAE/g), using a standard calibration curve established using serial dilutions of gallic acid (2–150 μg/mL).

The total flavonoid content (TFC) of the extracts was determined using the AlCl_3_ colorimetric assay following method of Nagy et al [15] using rutin as a standard. The absorbance was measured at 510 nm and TFC was expressed as mg rutin equivalent per gram of extract (mg RE/g), using a standard calibration curve established by measuring absorbance of serial dilutions of rutin (20–200 μg/mL). Both experiments were done in triplicate.

### Isolation of the major compounds from the root extract

The *n*-hexane fraction (3 g) was chromatographed over silica gel G column (4 cm x 60 cm) using gradient elution which started with *n*-hexane followed by addition of chloroform at 10% increments until 100% chloroform. Subsequently, solvent polarity was increased by addition of 10% increment of EtOAc (10%- 100%). Fractions (100 mL each) were monitored by TLC and similar fractions were pooled together to obtain 4 major fractions, PI-PIV. Fraction PI (0–30% chloroform in *n*-hexane) was subjected to column chromatography using silica gel G eluted with *n*-hexane followed by addition of 10% increments of chloroform till 100% (100 mL fraction each). Similar fractions were pooled and concentrated to yield compound **1** as white powder (50 mg). Compound **2** (white powder, 45 mg) was obtained after recrystallization from fraction PII (300 mg). Fraction PIV (700 mg) eluted with 50–100% EtOAc in chloroform, was subjected to column chromatography using silica gel eluted with a gradient of EtOAc (0–100%) in chloroform at 10% increments to give compound **3** (reddish yellow oil, 30 mg) and compound **4** (yellow oil, 20 mg).

The ethyl acetate fraction (6 g) was chromatographed on vacuum liquid column (7 cm x 15 cm) packed with silica gel H (200 g). Elution was carried out using chloroform followed by addition of EtOAc by 10% increment to 100%. Afterwards, methanol was added at 5% increments up to 25%. Fractions were monitored by TLC and pooled when appropriate to give 5 fractions EI-EV. Fractions EII and EIV were separately subjected to size exclusion chromatography using sephadex LH-20 eluted with methanol to afford isolation of compound **5** (yellow needles, 80 mg) and compound **6** (white needles, 170 mg), respectively.

Compound **3**: Yellow oil *m/z* 157.1037 for [M+H]^+^ calculated as C_10_H_21_O. ^1^H-NMR,400 MGHz *δ*_H_ ppm 0.93 (3H, m, H9), 0.94 (3H, m, H10), 0.97 (3H, m, H8), 0.97 (3H, m, H11),0.99 (3H, d, *J* = 6.9 Hz, H7), 1.23 (1H, br, H3), 1.92 (1H, dh, *J* = 2.5, 6.9 Hz, H6), 3.38 (1H, d, *J* = 2.5 Hz, H2), 3.42 (1H, d, *J* = 10.4 Hz, H5a), 3.54 (1H, d, *J* = 10.4 Hz, H5b).^13^C-NMR 100 MGHz *δ*_C_ ppm 16.5 (C9), 19.8 (C11), 20.3 (C8), 23.5 (C10), 23.6 (C7), 29.2 (C6), 29.6 (C3), 39.4 (C4), 73.3 (C5), 83.5 (C2).

Compound **4**: Yellow orange oil *m/z* 239.0992 corresponding to molecular formula C_10_H_16_NaO_5_^+^ for [M+Na]^+^. ^1^H-NMR,400 MGHz δ_H_ ppm 0.97 (3H, m, H7), 0.98 (3H, m, H8), 1.15 (3H, d, *J* = 5.6 Hz, H11), 2.03 (1H, m, H6), 2.55 (1H, m, H3), 3.79 (1H, dd, *J* = 10.9, 2.4 Hz, H5a), 4.09 (1H, dd, *J* = 10.9, 1.4 Hz, H5b), 4.74 (1H, t, *J* = 2.4 Hz, H2).^13^C-NMR 100 MGHz δ_C_ ppm 19.9 (C8), 20.3 (C11), 23.2 (C7), 28.8 (C6), 29.6 (C3), 34.5 (C3), 70.8 (C5), 79.3 (C2), .177.4 (C10), 178 (C9).

### HPLC analysis of the extracts

Chromatographic analysis was performed on a Dionex chromeleon apparatus equipped with Zobrax eclipse C8 column (4.6 mm X 250 mm, 5 μm) according to the method described previously [16]. The system was operated at column temperature of 30°C and flow rate of 1 mL/min using a binary gradient of 1% aqueous trifluroacetic acid, pH 2.5 (solvent A) and acetonitrile (solvent B). The chromatographic run consisted of two steps gradient: solvent B 5–85% over 40 minutes followed by a slower gradient to 100% B over 15 minutes and an isocratic elution at 100% B for 5 minutes. Eluted peaks were detected at 280 nm for phenolic acids and 360 nm for flavonoids. Each analysis was repeated three times. External standards were analyzed under the same conditions and standard calibration curves for gallic acid and myricetin were plotted using serial dilutions of each (20–200 μg/mL).

### Animals

Adult male Wistar Albino rats (180–200 g) were housed at 23 ± 2°C and 55 ± 5% humidity with 12 h light/dark cycle and fed on normal pellet rodent chow and water *ad libitum*. All experimental protocols were approved by the ethical committee for animals’ experimentation at Faculty of Pharmacy, Cairo University (July 13^th^, 2017, protocol No. MP 1432) and followed the Guide for the Care and Use of Laboratory Animals published by the U.S. National Institutes of Health (NIH Publication No. 85–23, revised 1996).

### Determination of median lethal dose (LD_50_)

For each extract, 20 rats were allocated into four groups of five rats per group [17]. Each group was orally dosed with a single dose of 100, 500, 1000 and 3000 mg kg^-1^ bwt aerial or root extract. The animals were observed over a period of 48 h for mortality, behavior and signs of toxicity including loss of appetite, vomiting, diarrhea or more than 10% weight loss.

### Experimental protocol for assessment of antidiabetic activity

Diabetes was induced in overnight fasted rats (groups **2**–**7**) by subcutaneous injection (s.c.) of streptozotocin (STZ, 50 mg kg^-1^ bwt in 0.1 M citrate buffer, pH 4.5) [18]. After one-week, rats with postprandial blood glucose > 250 mg/dL were selected for the study. Each extract was solubilized in 0.5% aqueous solution of carboxymethyl cellulose (CMC). Rats were randomly divided into seven groups (10 animals each). Group **1** (NC): Normal rats treated with both vehicles (0.1 M citrate buffer, pH 4.5, s.c.) once at the beginning of the experiment and daily oral dose of 0.5% CMC for 21 days. Group **2** (DC): Diabetic rats treated with daily oral dose of 0.5% CMC for 21 days. Groups **3** and **4**: Diabetic rats treated with a daily dose of 250 and 500 mg kg^-1^ bwt of REE, respectively. Groups **5** and **6**: Diabetic rats treated with a daily dose of AEE at 250 and 500 mg kg^-1^ bwt, respectively. Group **7**: Diabetic rats treated with a daily dose of metformin (100 mg kg^-1^ bwt) [19].

### Blood glucose level monitoring

Blood was collected after overnight fasting on day 0, 7, 14 and 21 of the experiment from the tail vein. Fasting blood glucose (FBG) was measured using One Touch ULTRA ®Glucometer (LifeScan, Johnson and Johnson, USA). At the end of the experiment, percentage reduction of the glucose levels at the 21^th^ day was calculated using the following formula:

$$Reduction\;in\;glucose\;level=\frac{FBG21-FBG0}{FBG0}\;X\;100.$$

Where *FBG21* = glycemic value after 21-day treatment, *FBG0* = value at induction.

It is denoted as change ^(A)^%.


$$reduction\;in\;glucose\;level\;at\;day\;21=\frac{FBGt-FBG[DC]}{FBG[DC]}\;X\;100.$$

Where *FBGt* = glycemic value of treated groups, FBG*[DC]* = glycemic value of diabetic group. It is denoted as Change ^(B)^%.

### Blood collection and tissue sampling

At the end of treatment, animals fasted overnight and blood was collected from retro-orbital plexus into serum preparation tube [20]. The separated sera were divided into aliquots and stored at -20°C for insulin assay. The animals were directly decapitated under mild anesthesia after collection of blood samples. At autopsy, pancreases were removed and cleaned from connective tissues, washed in ice-cold 1.15% KCl, and blot dried separately. Pancreases (n = 5) from each group were stored in 10% formalin solution for further histopathological examination. Remaining pancreases (n = 5) were kept at -80°C for Glut2 and Glut4 assays.

### Measurement of serum insulin level

Serum samples were analyzed for insulin using ELISA kit; (kit ref. Rat insulin, Catalog No. MBS724709, San Diego, CA 92195–3308, USA; Sensitivity, 0.1 ng/mL). Experimental procedures followed manufacture’s protocol and the kits used have a precision: intra-assay CV (%) and interassay CV (%) less than 10%.

### Measurement of Glut2 and Glut4 expressions

Total RNA was extracted from pancreatic tissue samples using Qiagen tissue extraction kit (Qiagen, USA) according to manufacturer’s instructions. RNA purity was determined using dual wavelength spectrophotometer (Beckman, USA) and its integrity was examined by running through 1.5% denatured agarose gel. The extracted RNA (0.5–2.0 μg) was converted to cDNA using reverse transcription kit (Fermentas, USA) according to manufacturer’s instructions. Three microliters of random primers were added to the 10 μg of RNA. The total volume was adjusted up to 31 μL with DEPC-treated water then incubated in Bio-Rad T100 TM thermal cycler at 65°C for 10 min. The master mix cDNA was prepared by mixing 5 μL of first strand buffer, 2 μL of 10 mM dNTPs and 1 μL Moloney Murine Leukemia Virus (M-MuLV) reverse transcriptase (50 U/μL). Total volume was adjusted to 19 μL by adding DEPC water to complete volume of master mixture to 50 μL. The mixture was incubated in the programmed thermal cycler for one hour at 37°C followed by inactivation step at 95°C for 10 minutes, and finally cooled at 4°C. Then, RNA changed into cDNA and the converted cDNA were stored at –20°C for use in real time qPCR.

Real-time qPCR amplification was performed using an Applied Biosystem with software version 3.1 (StepOne™, USA) with the primer sets optimized at the annealing temperature (primer sequences are shown in **Table 1**). For each sample, 1.0 μL forward primer, 1.0 μL reverse primer, 12.5 μL Syber green mix, 5.0 μL cDNA template, and 5.5 μL RNAse free water were added. Running condition for RT-PCR were set as 1 cycle of 2 min at 50°C and 40 cycles of 15 s at 95°C, 60 s at 60°C and 60 s at 72°C. *β-*Actin mRNA was used as a reference. PCR products were photographed and subjected to band intensity analysis using Applied Bio system software. The method of 2^–ΔΔCt^ was adopted for analysis.

**Table 1:** The primer sequence of the studied genes.

Target gene	Primer sequence
Glut4	Forward primer: 5’-CAACTGGACCTGTAACTTCATTGT-3’ Reverse primer: 5’-ACGGCAAATAGAAGGAAGACGTA-3
Glut2	Forward primer:5’-AAGGATCAAAGCCATGTTGG -3` Reverse primer:5′-GGAGACCTTCTGCTCAGTGG-3`
Beta actin	Forward primer:5′-CAG GAT GGC GTG AGG GAG AGC-3′ Reverse primer: 5′-AAG GTG TGA TGG TGG GAA TGG-3′

### Histopathological examination of pancreas

Pancreas tissues were fixed in 10% formalin saline for 24 hours then washed in tap water followed by washing in serial dilutions of alcohol for dehydration. Specimens were cleared in xylene and embedded in paraffin at 56°C in hot air oven for 24 h. Paraffin bees wax tissue blocks were prepared for sectioning at 4 microns thickness by sledge microtome. The obtained tissue sections were collected on glass slides, deparaffinized, and stained by hematoxylin & eosin stain for routine examination through light electric microscope [21].

### DPPH radical scavenging assay

DPPH free radical scavenging assay was performed as described previously [22]. DPPH in methanol (0.02 mg/mL) was incubated with different concentrations of AEE and REE for 30 min in dark and the reduction in color was measured at 517 nm using spectrophotometer. The percentage of DPPH radical scavenging (I) was calculated as follows

$$I=(1-\frac{Ac}{At})\times 100$$

Where **At** = absorbance of the tested extracts, **Ac** = absorbance of control (DPPH solution without the tested extract). Ascorbic acid was used as a standard antioxidant and showed IC_50_ of 8.5 μg/mL.

### α-glucosidase inhibitory assay

α-Glucosidase enzyme inhibitory activity was determined using the method reported by You et al. [23]. Serial dilution of REE and AEE (7.81–1000 μg/mL) were incubated with 500 μL of 1 U/mL α-glucosidase solution. *p*-Nitrophenyl glucopyranoside (*p*-NPG), 5 mM was used as a substrate. Released *p*-nitrophenol was estimated by measuring absorbance at 405 nm. Acarbose was used as a positive control at the same concentrations.

The percentage of inhibition was calculated using the following formula:

$$\%\;inhibition=(1-\frac{Abs\;control}{Abs\;sample})\times 100$$

Where: **Abs control**: absorbance of the control reaction (containing all reagent except the test sample); **Abs sample**: absorbance of the test sample.

Each experiment was performed in triplicate and concentrations that caused 50% inhibition (IC_50_) were calculated from response curve.

### α-amylase inhibitory assay

Inhibition of α-*amylase* enzyme was determined using the method reported by Narkhede et al. [24].

Briefly, 1 mL of the REE and AEE of various concentrations (7.81–1000 μg/mL) and 1 mL of enzyme solution (0.5 mg/mL) were mixed together and incubated at 25°C for 10 min. Then, 1 mL of starch solution (0.5%) was added and the mixture was further incubated at 25°C for 10 min. The reaction mixture was then stopped by adding 2 mL of dinitrosalicylic acid (DNS) followed by heating the reaction mixture in a boiling water bath (5 min). After cooling, the absorbance was measured at 565 nm. The percentage of inhibition was calculated as mentioned for α-glucosidase assay.

### Molecular docking

Molecular docking studies were performed using Molecular Operating Environment MOE platform, 2015.1. (Chemical Computing Group, Canada). 3D structures of the tested compounds were introduced using the builder interface of MOE. Partial charges were added and energy minimization was performed using MMFF94x force field. The structure of human glycerol-3-phosphate dehydrogenase, GPDH, co-crystallized with dihydroxy acetone phosphate (DHAP) was downloaded from protein databank, code 1WPQ, (https://www.rcsb.org). After hydrogen addition and energy minimization, the co-crystallized ligand was removed and redocked to RMSD 0.8591 Å. Molecules were docked in the active sites of GPDH using triangle matcher placement method and their docking score was calculated using London dG function.

### Statistical analysis

All data were represented as mean ± standard error (SE); the results were analyzed by One-Way (ANOVA), with post hoc Tukey test. Differences were considered statistically significant at *P* < 0.05. The data analyses were conducted using GraphPad Prism7 (GraphPad Software, San Diego CA, USA).

### Results and discussion

The LD_50_ study carried on rats revealed no mortality among animals and no signs of toxicity up to 3000 mg kg^-1^ bwt of both extracts. Animals that received streptozotocin (STZ) showed clear hyperglycaemia manifested by FBG > 400 mg/dL. At day-21, treatment with REE at high and low doses (500 and 250 mg kg^-1^ bwt) exhibited a significant decrease in glucose level by -44%, and -39%, respectively, compared to control diabetic group (DC). Furthermore, treatment with AEE at high dose and low dose (500 and 250 mg kg^-1^ bwt) significantly diminished glucose level by -41% and -37%, respectively. Compared to the groups at their respective day zero, treatment with REE at high and low doses (500 and 250 mg kg^-1^ bwt) exhibited a significant decrease in glucose level by -31.48%, and -7%, respectively. Moreover, treatment with AEE at high dose and low dose (500 and 250 mg kg^-1^ bwt) significantly reduced glucose level by -5.51% and -6.88%, respectively **(Table 2)**. Collectively, REE at a dose of 500 mg kg^-1^ bwt was most effective in attenuating glycaemic status than other investigated treatments except the standard drug metformin (MF).

**Table 2:** Effect of REE, AEE and metformin on serum glucose concentration (mg/dL) in diabetic adult male rats.

Group	Zero-	Day-7	Day-14	Day-21	Change^A^%	Change^B^%
NC	78.13 ± 8.32	79.88 ± 7.45	85.88 ± 8.0	87.50 ± 7.2		
DC	429.40 ± 86.4	438.2 ± 85.3	465.6 ± 60.6	527.0 ± 58.7	22.73	
DC + MF	319.0 ± 117.1	262.33 ± 127.0	221.83 ± 114.9	181.67 ± 29.3^a^*^b^***	-43.05	-66%
DC + REE (500)	428.33 ± 83.1	375.33 ± 74.2	331.67 ± 58.7	293.5 ± 55.^a^*^b^**	-31.48	-44%
DC + REE (250)	350.25 ± 133.6	310.24 ± 137.9	335.36 ± 178.9	323.68 ± 178.3^b^**	-7.59	-39%
DC + AEE (500)	356.0 ± 125.3	285.2 ± 115.2	264.6 ± 105.6	312.0 ± 127.6^b^*	-5.51	-41%
DC + AEE (250)	357.43 ± 131.57	347.43 ± 131.2	308.0 ± 94.9	332.83 ± 86.5^b^*	-6.88	-37%

^a^; represent significance from their respective controls at zero-time

^b^; represent significance from diabetic group (DC) at 21-day.

*p < 0.05

**p < 0.01 and

***p < 0.001

Animals group are designated as: **NC**: normal control, **DC**: diabetic control, **DC** + **MF**: diabetic animals that received daily dose of metformin 100 mg kg^-1^ bwt, **DC + REE**: diabetic animals that received daily dose of root ethanol extract, **DC + AEE**: diabetic animals that received aerial part ethanol extract.

Change^(A)^: change in glycemic value in treated groups at day 21 from its respective day zero.

Change^(B)^: Change in glycemic value in day 21 from the value in diabetic group (DC) in day 21.

In attempt to further explore the basis for the antidiabetic action of both extracts, level of serum insulin as well as expression levels of two glucose transporters Glut2 and Glut4 in the pancreatic tissue were estimated. Glucose transporters are specific facilitative glucose carrier proteins including glucose transporter 2 and 4 (Glut2 and Glut4) which are expressed in liver, intestine, kidney, and *β*-pancreatic islet cells [25]. In the current study, severe depletion in serum insulin and down regulation in Glut2 and Glut4 were observed in diabetic animals as low levels of these transporters will limit uptake of circulating glucose into pancreases and other tissues causing decreased insulin secretion and hyperglycaemia [26]. When compared to metformin (MF) which exerts its antidiabetic effect by increasing glucose consumption and insulin sensitivity, administration of REE at both doses augmented insulin level and up-regulated expression of Glut2 and Glut4 to comparable levels (**Table 3**). Meanwhile, administration of AEE at the same dose showed only negligible increase in insulin level, Glut2 and Glut4 expression further confirming variability in the antidiabetic effect of both extracts which appeared to be mediated, at least partially, by increased glucose uptake in case of REE.

**Table 3:** Effect of REE and AEE on insulin level and glucose transport in diabetic rats.

Group	Insulin (μU/mL)	Glut 2	Glut4
NC	80.69 ± 8.65	1.01 ± 0.02	1.02 ± 0.03
DC	28.91 ± 2.12^a^***	0.18 ± 0.09 ^a^***	0.20 ± 0.02^a^***
DC + MF	52.16 ± 7.96 ^a^***^b^***	0.72 ± 0.13 ^b^***	0.63 ± 0.06^a^***^b^***
DC + REE (500)	62.06 ± 3.71 ^a^*^b^***	0.83 ± 0.08^b^***	0.64 ± 0.06^a^**^b^***
DC + REE (250)	54.76 ± 5.76 ^a^***^b^***	0.83 ± 0.05 ^b^***	0.69 ± 0.05 ^a^***^b^***
DC + AEE (500)	31.99 ± 2.36 ^a^***^c^**	0.19 ± 0.11 ^a^***^c^***	0.23 ± 0.05 ^a^***^c^***
DC + AEE (250)	36.41 ± 4.56^a^***^c^*	0.22 ± 0.17^a^***^c^***	0.23 ± 0.06 ^a^***^c^***

^a^; Significance from their respective controls at zero time.

^b^; significance from diabetic group.

^c^; significance from reference drug (metformin)

*p < 0.05

**p < 0.01 and

***p < 0.001

**Glut2**: glucose transporter-2, **Glut4**: glucose transporter 4. Animals group are designated as: **NC**: normal control, **DC**: diabetic control, **DC + MF**: diabetic animals that received daily dose of metformin 100 mg kg^-1^ bwt, **DC + REE**: diabetic animals that received daily dose of root ethanol extract, **DC + AEE**: diabetic animals that received aerial part ethanol extract.

**Fig 1:**
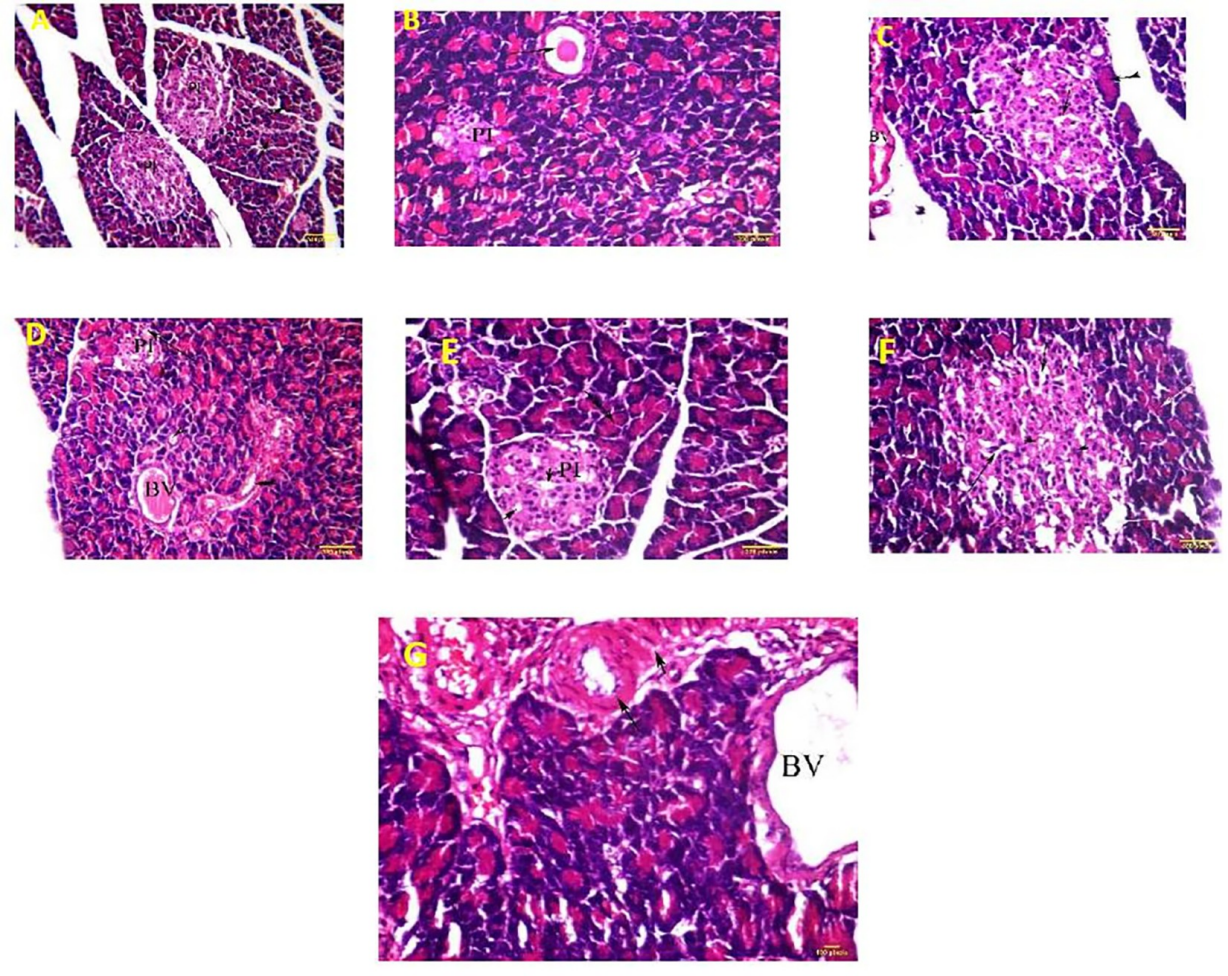
Photomicrographs of rat pancreases isolated from different animal groups. Photomicrographs of cross section of the pancreatic tissue of **A**: normal healthy animals showing normal pancreatic islets (PI) and pancreatic acini (arrow), **B**: diabetic group showing pancreatic duct inspissiated (arrow), pancreatic islet (pi) with sever reduction in size and hypocellularity. **C**: animals treated with REE 250 mg/kg showing: pancreatic islet (short arrow), inspissated pancreatic duct (long arrow) and blood vessel (double arrow) and pancreatic acini (arrow head). **D**: animals treated with REE 500 mg kg^-1^ bwt showing pancreatic islet moderate in size and dilated congested blood vessel (arrow). **E:** animals treated with 250 mg kg^-1^ bwt AEE showing pancreatic blood vessels with thickened walls (arrow) and perivascular fibrotic reaction (short arrow). **F**: animals treated with AEE (500 mg kg^-1^ bwt) showing islets with normal size and vaccuolation between cells (arrowhead), pancreatic duct (arrow). (H & E). **G**: Animals treated with metformin showing: pancreatic islets with vacuolation between cells (arrow), dilated congested blood vessel (BV), and intact pancreatic acini (double arrow).

Histopathological examination was conducted on the pancreatic tissue in various animal groups. Diabetic untreated animals showed many pathological alterations in the pancreatic tissue compared with non-diabetic animals manifested by severe reduction in the number and size of pancreatic islets with hypocellularity, dilated congested capillaries and mild vacuolation in and between cells (**Fig 1A and 1B**). Significant improvement was observed in the group treated with MF, where many pancreatic islets were normal in size and cellularity **(Fig 1C).** In the groups treated with REE at both doses, only few cells displayed atrophies with degenerative changes and mild hypocellularity was observed (**Fig 1C and 1D**). Meanwhile, animals treated with high dose of AEE showed mild to moderate pathological changes, where pancreatic islets with dilated capillaries and vacuolation were observed and occasional islets displayed reduction in size (**Fig 1E**), while lower dose of AEE failed to produce significant amelioration of tissue damage (**Fig 1F**).

Moreover, *Limonium axillare* root extract (REE) was highly enriched in phenolic compounds with total phenolic content of 252 mg GAE/g of extract compared to 124 ± 2.129 mg GAE/g for AEE. Meanwhile, AEE showed higher TFC (26.6 mg QE/g) than REE (**Table 4)**. This resulted in a good *in vitro* radical scavenging activity of REE (IC_50_ = 25.2 μg/mL) compared to much weaker activity of AEE with IC_50_ of 407.9 μg/mL explaining to a certain extent the preservation of pancreatic tissue integrity in animals receiving REE rather than AEE. STZ is thought to induce diabetes by liberation of superoxide radicals which then cause DNA damage and subsequent *β*-cells necrosis [18]. Therefore, strong radical scavenging activity of the extract can ameliorate its detrimental effect.

**Table 4:** Chemical characterization of REE and AEE and their ethyl acetate fractions.

Compounds	Root		Aerial parts	
	Ethanol Ext	EtOAc Fr.	Ethanol Ext	EtOAc Fr.
Total phenolics^a^	252.6 ± 0.04	nd	124 ± 0.22	nd
Total flavonoids^b^	20.326 ±0.023	nd	26.6 ± 0.0819	nd
Gallic acid^c^	3.46 ± 0.05	9.026 ± 0.01	20.6 ± 0.03	8.5 ± 0.005
Myricetin^c^	5.6 ± 0.014	7.85 ± 0.002	10.0 ± 0.004	6.0 ± 0.001
Umbelliferone^c^	10.0 ± 0.008	6.35 ± 0.001	1.30 ± 0.001	-

EtOAc Fr = Ethyl acetate fraction, Ext = extract, nd = not determined

^a^: mg GAE/g

^b^: mg RE/g

^c^:μg/mL

Values are expressed as means ± S.E., n = 3

Additionally, REE, AEE and their fractions were evaluated for possible inhibition of α-glucosidase and pancreatic α-amylase, two enzymes involved in releasing glucose from dietary carbohydrates. Among investigated samples, REE, its EtOAc fraction and AEE showed the most potent inhibition of α-glucosidase enzyme with IC_50_ values at 44.8, 59 and 60.4 μg/mL, respectively, compared to acarbose (IC_50_ = 30.57 μg/mL), **Table 5**. Similar results were obtained in pancreatic α-amylase inhibition assay, where *n*-hexane fraction of REE and EtOAc fractions of both REE and AEE showed strong inhibition of α-amylase with IC_50_ values at 41.6, 45.8 and 48.1 μg/mL, respectively (IC_50_ of acarbose was observed at 34.71 μg/mL), **Table 5**. These findings indicate that inhibition of these enzymes may be a contributing mechanism in the antidiabetic activity of both extracts. It is worth mentioning that several *Limonium* species have shown strong *in vitro* inhibitory activity against both α-glucosidase and pancreatic α-amylase [27, 28].

**Table 5:** α-glucosidase and α-amylase inhibitory activity of REE and AEE and their fractions measured as IC_50_ (μg/mL).

Sample		IC_50_ α-glucosidase	IC_50_α-amylase
**REE**	Total extract	44.8	89.3
	*n*-hexane Fr.	89.3	41.6
	DCM Fr.	426.04	192.13
	EtOAc Fr.	59.0	45.8
	*n-*Butanol	229.3-	115.9
**AEE**	Total extract	60.4	115.99
	*n*-hexane Fr.	123.7	NA
	DCM Fr.	341.9	111.6
	EtOAc Fr.	192.2	48.13
	*n-*Butanol	47.7	805.9

DCM: dichloromethane, EtOAc: ethyl acetate, Fr: fraction, NA: not active

**Fig 2:**
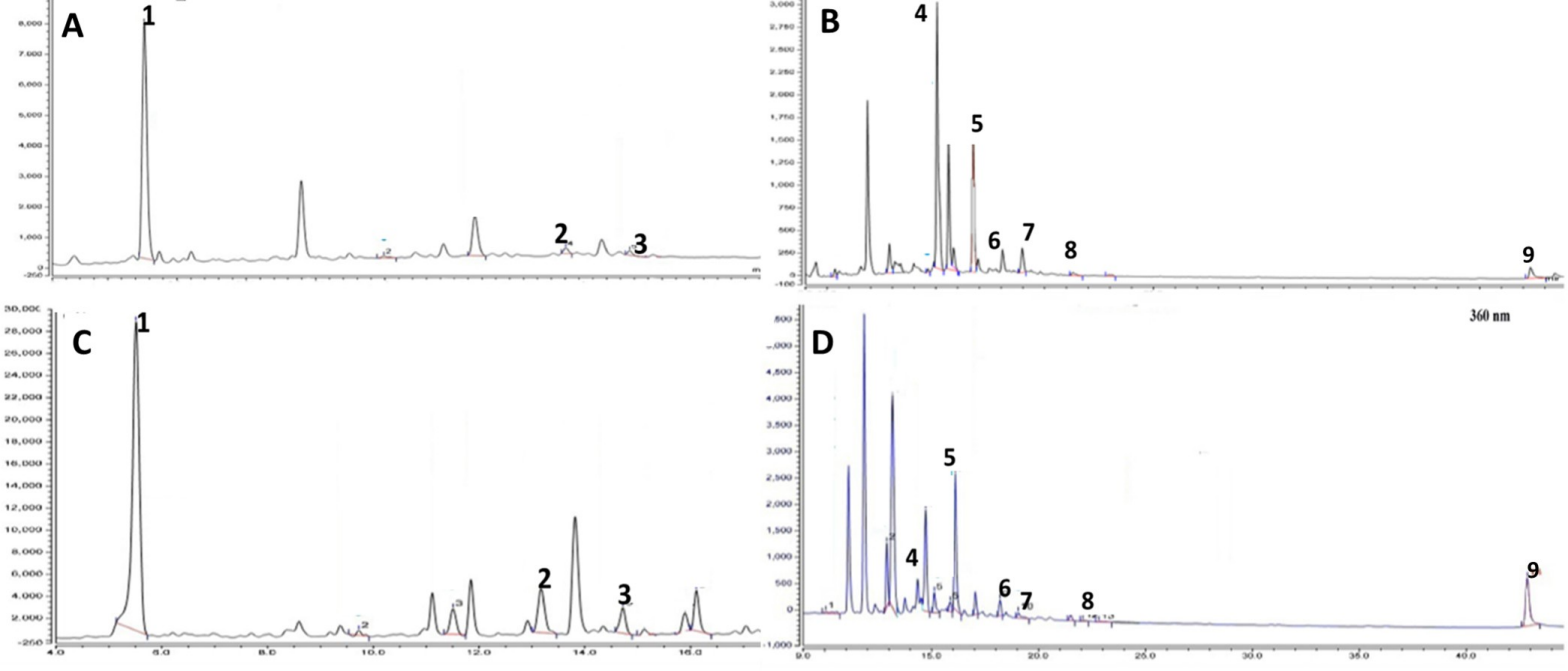
HPLC chromatograms for AEE and REE at λ = 280 and 360 nm. **A** and **B**:HPLC analysis for REE showing eluted compounds with UV absorbance at λ = 280 and 360 nm, respectively. **C** and **D**: HPLC chromatogram of eluted compounds detected at λ = 280 and 360, respectively. Identified compounds are labeled as **1**: gallic acid, **2**:ellagic acid, **3**: ferulic acid, **4**: umbelliferone, **5**: myricetin, **6**:quercetin, **7**:coumarin, **8**:apigenin and **9**:kaempferol.

HPLC analysis of both extracts using external standards previously isolated from the plant identified umbelliferone and gallic acids as the major constituents in REE (**Fig 2**), while AEE was enriched with the flavonoid myricetin in addition to gallic acid. Myricetin and its glycosides have been reported in other *Limonium* species including *L*. *gmelinii* and *L*. *bicolor* [29, 30]. Moreover, myricetin was found to accumulate in roots of *L*. *bicolor* in response to salt stress [31] Literature has shown that leaves of different amaranth species, such as *A*. *gangeticus*, drought-tolerant leafy vegetable amaranth, and *A*. *tricolor* had abundant gallic acids [32–34]. Similarly, leaves of salt-tolerant vegetable amaranth, *A*. *tricolor*, drought-tolerant vegetable amaranth, and red amaranth had abundant myricetin which is corroborative to the present findings [35, 36]. Other identified metabolites included ellagic acid, ferulic acid, kaempferol, quercetin, coumarin and apigenin which all have been reported in the plant previously [12, 37] (**Fig 2**). Myricetin and quercetin are known inhibitors of both α-glucosidase and α-amylase partially explaining *in vitro* assay results and likely contributing to antidiabetic activity of the extract by diminishing the release of glucose from dietary carbohydrates [38]. Meanwhile, gallic and ellagic acids and flavonoids are potent antioxidants which can offset the detrimental effect produced by superoxide radicals released as a result of STZ intoxication [39–41].

**Fig 3:**
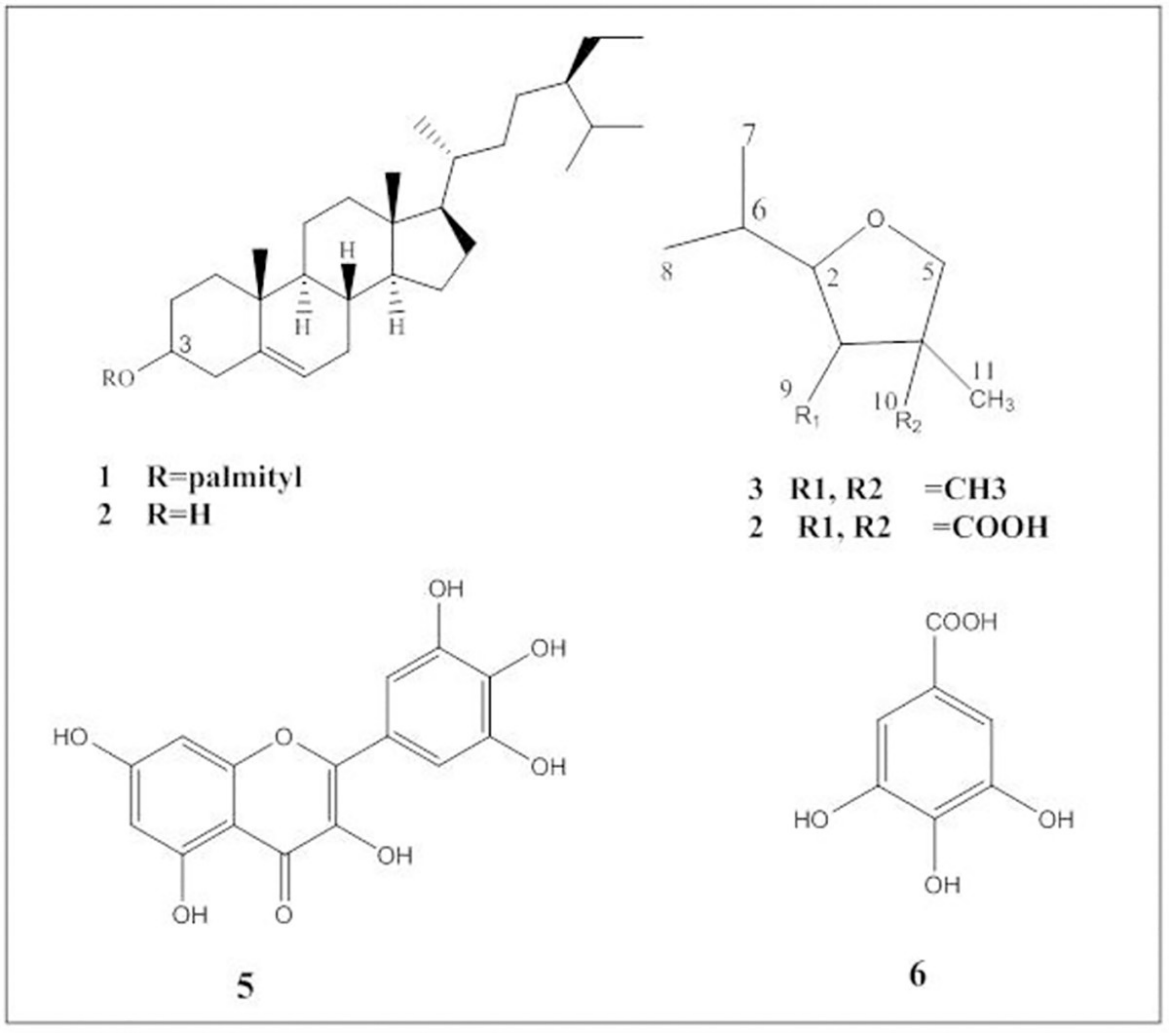
Compounds isolated from the root extract of *Limonium axillare*.

To follow up on the bioactive fractions, *n*-hexane and EtOAc fractions were subjected to repeated column chromatography which led to the isolation of six compounds including four known compounds, namely *β-*sitosterol-*3-O*-palimtate (**1**) and *β-*sitosterol (**2**) from *n*-hexane fraction and myricetin (**5**) and gallic acid (**6**) from EtOAc fraction (**Fig 3**). Additionally, two new compounds were isolated from the *n*-hexane fraction and are designated here as compounds **3** and **4.** All compounds were identified based on their chromatographic behaviours and 1D and 2D-NMR data. For compound **3**, ^1^H and ^13^C NMR spectra did not show any signals above *δ*_H_ 3.6 and *δ*_C_ above 84, respectively suggesting a tetrahydrofuran ring structure with no functional group substituents. ^1^H-^1^H-COSY and ^1^H-^13^C correlation spectra established the connectivity between various substituents as illustrated in **S1-S3 Figs in S1 File** and compound **3** was chemically identified as 1-isopropyl- 3,4,4, trimethyl-tetrahydrofuran (**Fig 3**). In higher plants, tetrahydrofuran-terpenes and sesquiterpenes are components responsible for aroma including several tetrahydrofuran predominately isolated from wine and grapes [42].

The ^13^C-NMR spectrum and MS analysis of compound **4** indicated the presence of two carboxylic groups *δ*_C_ 177.4 and 178, which resulted in downfield shifts of H2, H3 and H5. Analysis of 2D-correlation spectra (**S4-S6 Figs in S1 File**) identified compound **4** as 2-isopropyl-4-methyltetrahydrofuran-3,4-dicarboxylic acid, which is reported for the first time (**Fig 3**).

**Fig 4:**
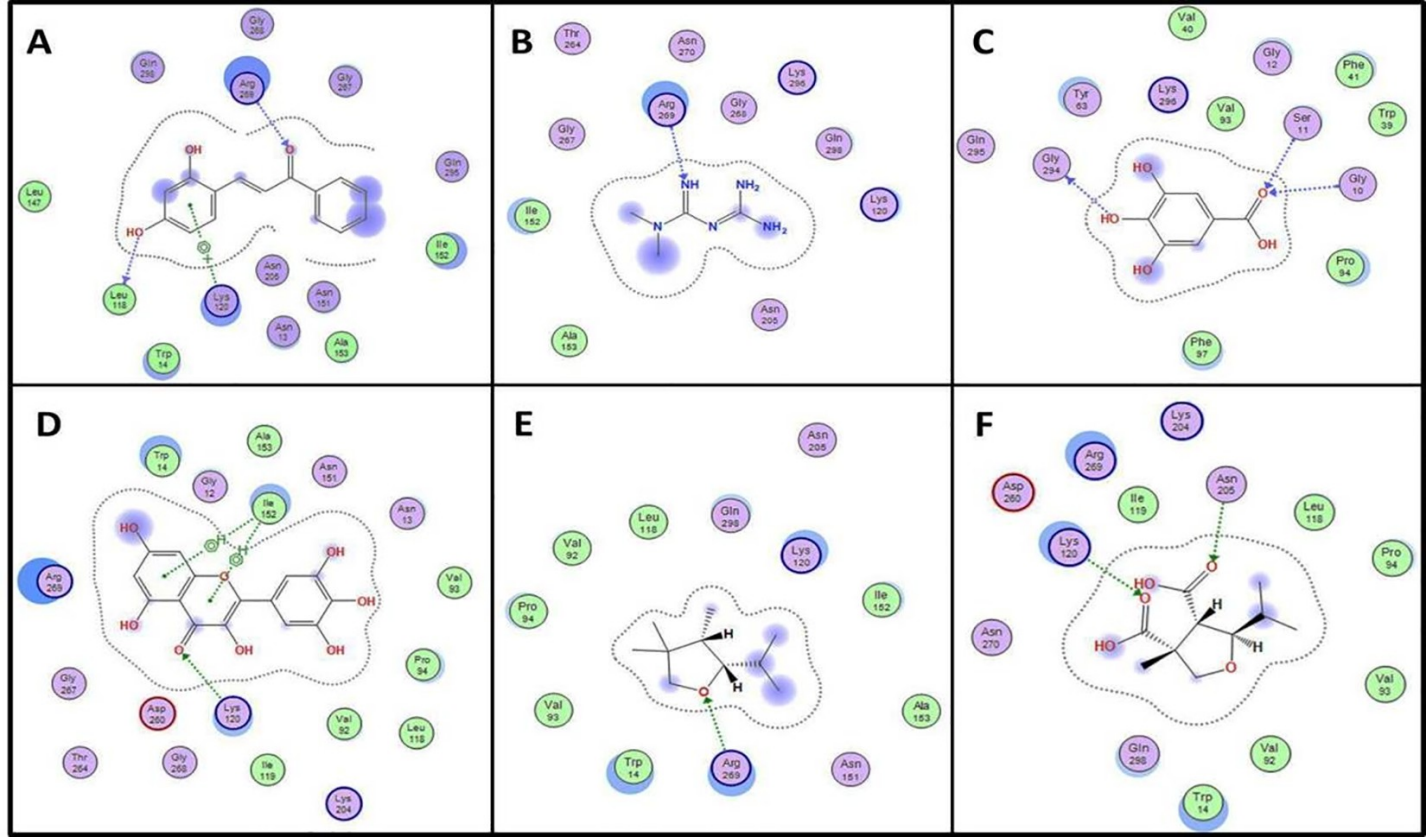
Enzyme ligand interactions of *Limonium axillare* phytoconstituents in the active site glycerol phosphate dehydrogenase. Diagrams representing 2D enzyme ligand interaction in the active site of GPDH with tested compounds A: 2’,4’ dihydroxychalcone, B: metformin, C: gallic acid, D: myricetin, E: compound **3**, F: compound **4**.

Finally, we used molecular docking to investigate possible affinity of major REE constituents to glycerol-3-phosphate dehydrogenase GPDH, a newly identified target for metformin which inhibition leads to suppression of gluconeogenesis [43]. Potential inhibitors that were identified for GPDH include chalcone derivatives and some flavonoids which showed good inhibitory activity for bacterial and protozoal GPDH [44, 45]. Docking of 2’,4’ dihydroxychalcone in the active site of human GPDH revealed a key hydrogen bond interaction with Arg269, which is a key residue in interacting with the phosphate group of its substrate dihydroxy acetone phosphate (DHAP). Other interactions of dihydroxychalcone included two other key amino acid residues Lys120, which stabilizes the hydroxyl and carbonyl oxygen of DHAP and Leu118, which is involved in interaction with NAD [46]. Among the compounds isolated from REE, myricetin, showed better docking score than dihydroxychalcone (**Table 6**) with possible interaction with Arg269, Leu118, Lys120 and Ile152. Metformin, compound **3** and compound **4** showed at least one interaction with these key amino acid residues (**Fig 4)**.

**Table 6:** Molecular docking results and ligand inteactions of major phytoconstituents of *Limonium axillare* root extract in active site of glycerol-3-phosphate in comparison with known inhibitors.

Ligand	Docking score kcal/mol	Residue	Type of interaction
**2’,4’Dihydroxychalcone**	-6.2652	Leu118	H-bond
		Lys120	H^+-^arene
		Arg269	H-bond
**Metformin**	-4.6791	Arg269	H-bond
**Gallic acid**	-4.903	Gly10	H-bond
		Ser11	H-bond
		Gly294	H-bond
**Myrecitin**	-6.6121	Lys120	H-bond
		Ile152	H^+-^arene
**Compound 3**	-5.6075	Arg269	H-bond
**Compound 4**	-5.7992	Lys120	H-bond
		Asn205	H-bond

Based on the chemical profiles and biological assessment of both REE and AEE extracts, the ethanobotanical use of the roots of *Limonium axillare* seem to be justified. While extract of the aerial part showed mild antidiabetic activity, likely mediated through inhibition of α-glucosidase and α-amylase, antidiabetic action of root extract may be mediated through multiple mechanisms including antioxidant activity, enhanced insulin secretion, in addition to marked increase in expression of Glut2 and Glut4 and therefore increasing glucose uptake. We also provided evidence from *in silico* studies to suggest that constituents of root extract may have binding affinity to glycerol-3-phosphate dehydrogenase. The study encourages further investigation of the antidiabetic effects of *L*. *axillare* as it is likely to be affected by the plant habitat and its geographical origin. Also, it should be noted that other underlying mechanism for antidiabtic activity may also be involved. It is not clear from the current study, if the anti-hyperglycemic action of *L*. *axillare* can be sustained over a period of time and whether it can prevent other complications of DM, which ought to be addressed in future studies. We would like to point out that under-investigated flora are potential repertoires of novel natural products with possible biological activities and our study underscores the importance of experimental investigations of unexplored ethnobotanical remedies either by classic phytochemical investigation or through comprehensive metabolomics studies.

## Supporting information

S1 File NMR spectra for both compound 3 and 4. (DOCX)
