# Supplementary material for: Chemical and biological investigations of Limonium axillare reveal mechanistic evidence for its antidiabetic activity
Source: PLoS One. 2021 Aug 6;16(8):e0255904. doi: 10.1371/journal.pone.0255904 (PMC8345833; doi:10.1371/journal.pone.0255904)

**S1 File**

**NMR spectra of compounds 3 and 4**

**Figure S1: Key HSQC cross peaks observed in the spectrum of compound 3, and their assignment as recorded in CDCl3**


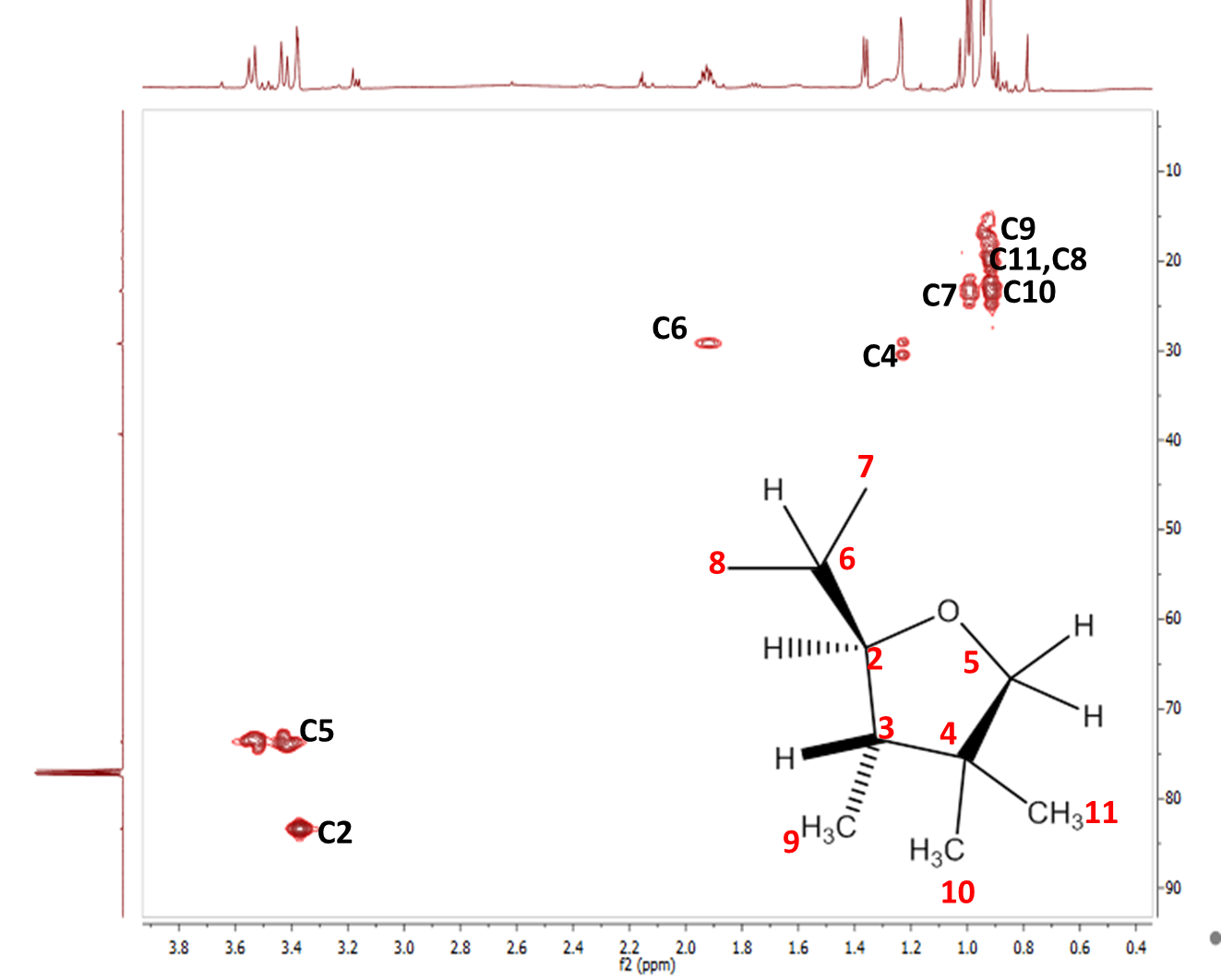


**Figure S2: Key cross peaks identified in the HMBC spectrum and key H-C correlations for compound 3 recorded in CDCl3**


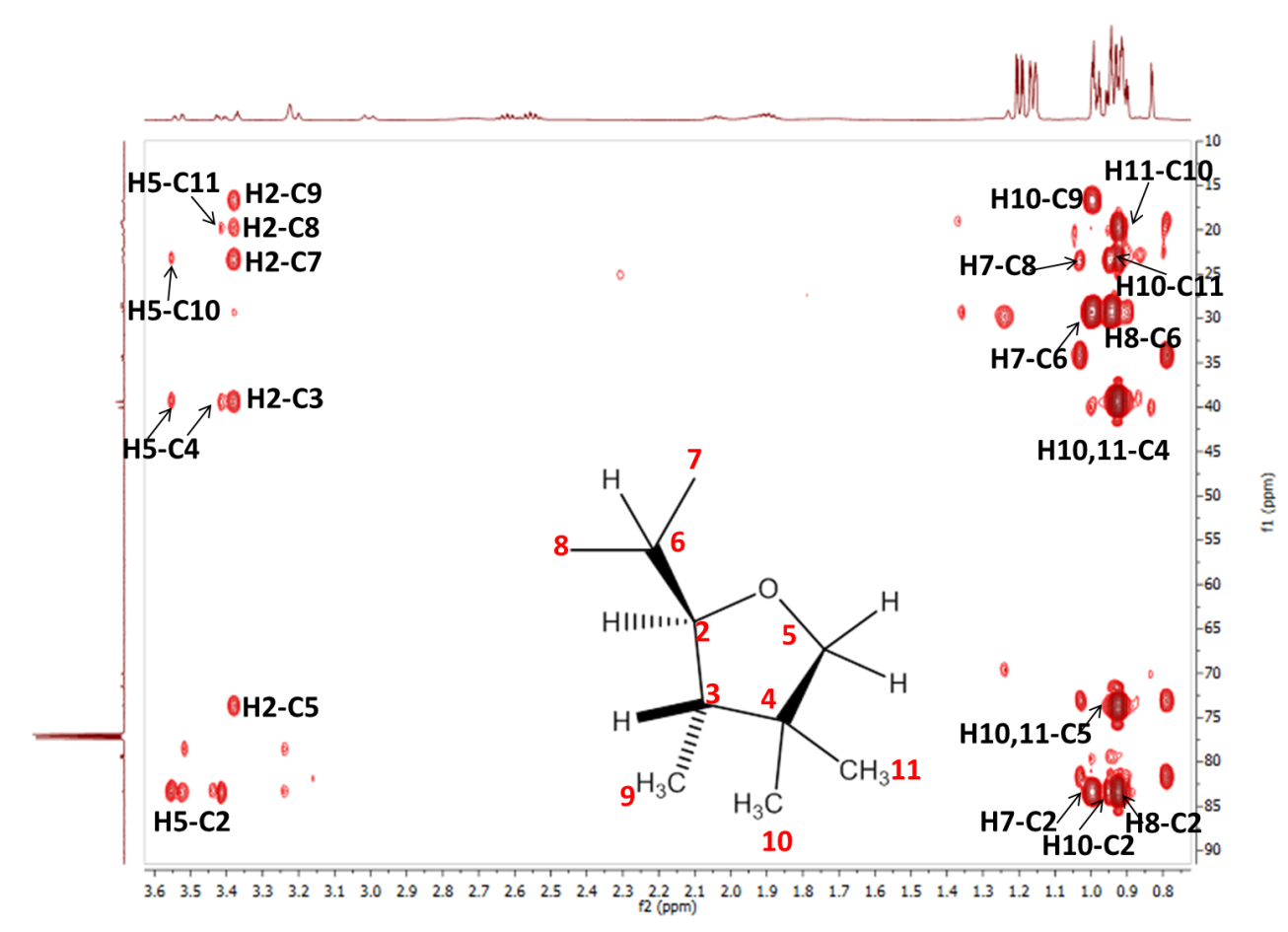


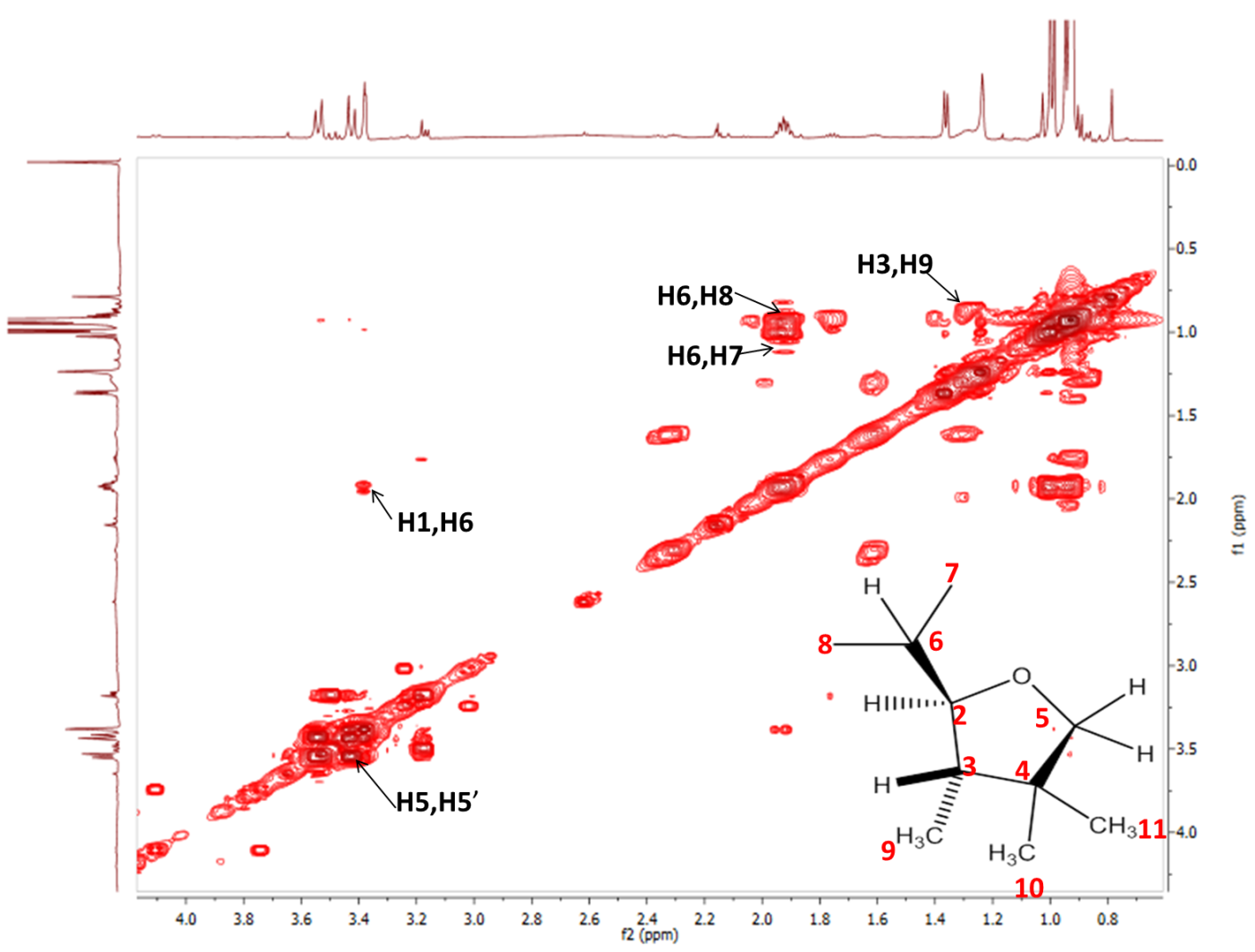

**Figure S3: Key H-H correlations in the COSY spectrum of compound 3 recorded in CDCl3**

**Figure S4: KEY cross peaks in the HSQC spectrum of compound 4 recorded in CDCl3**


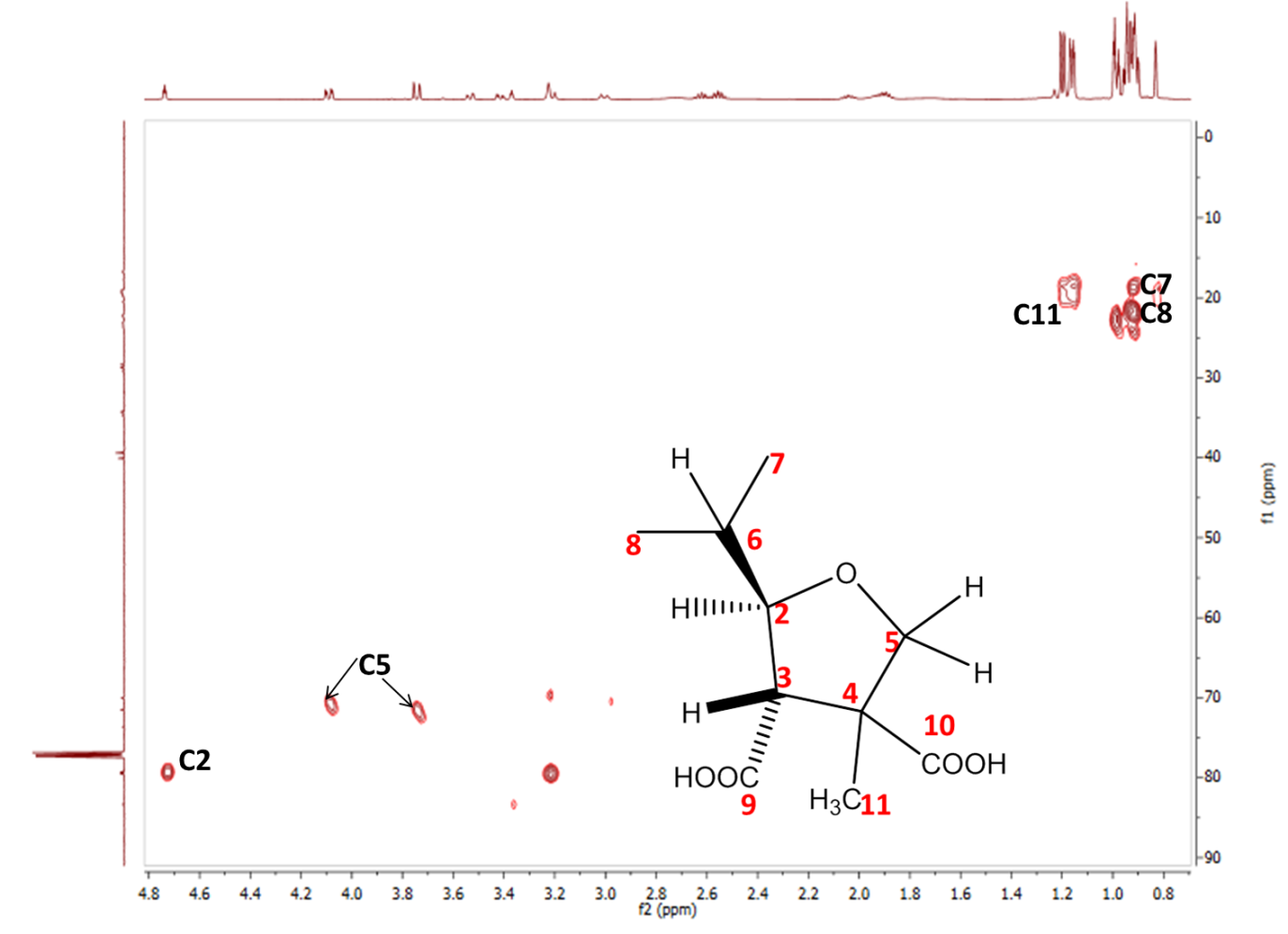


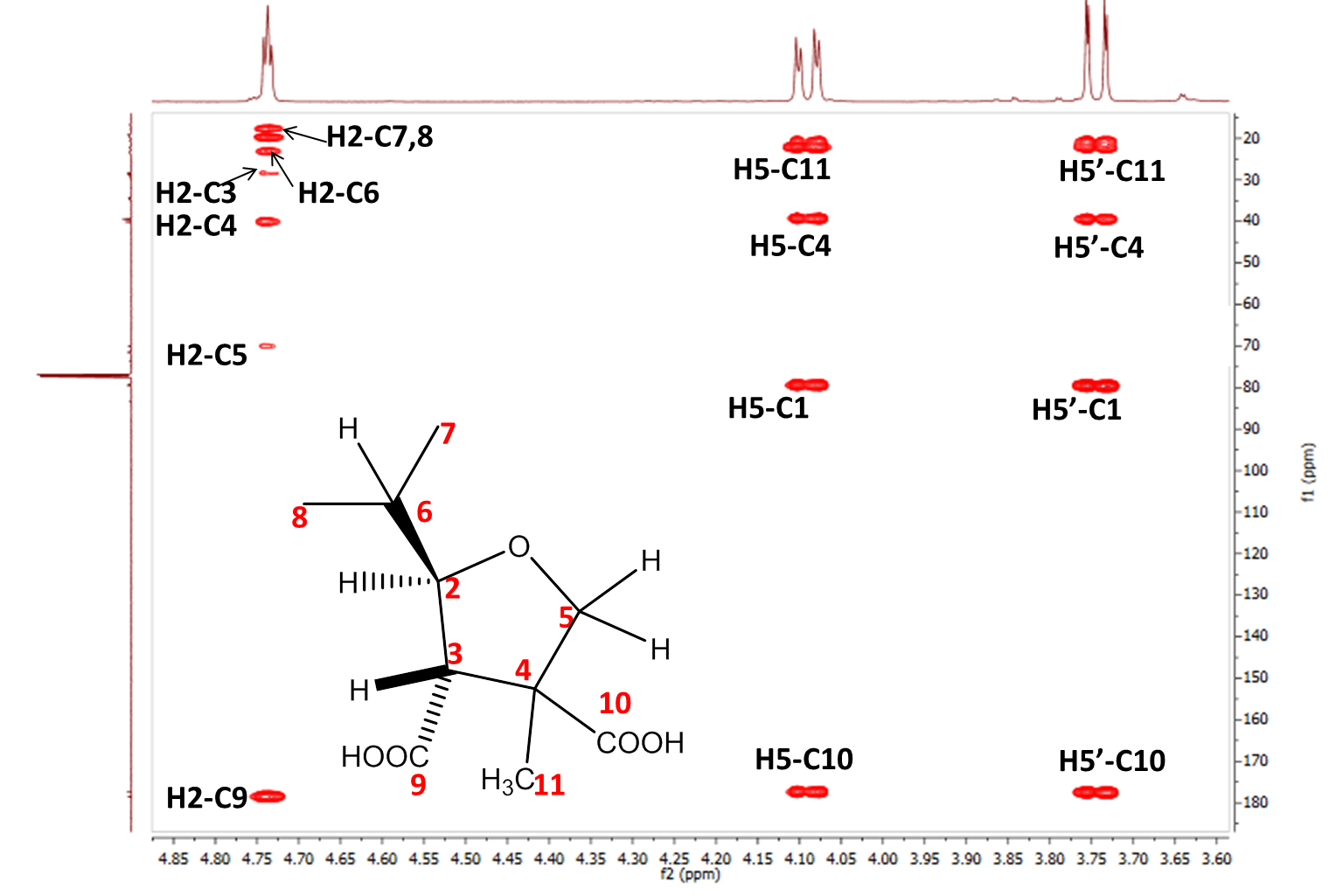


**Figure S5: Key cross peaks in the HMBC spectrum of compound 4 recorded in CDCl3**

**Figure S6: key H-H correlation in the COSY spectrum of compound 4 observed in CDCl3**
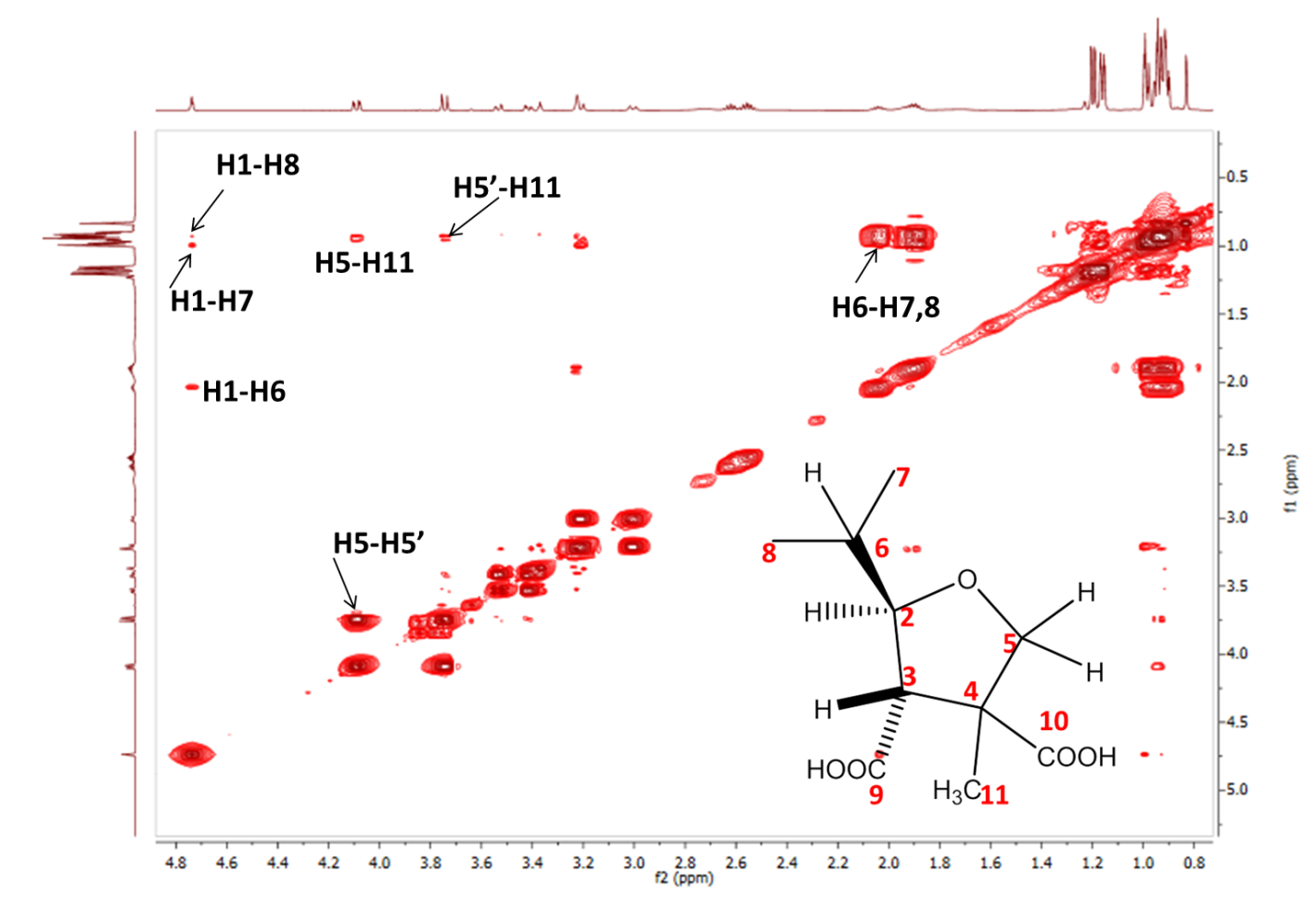

Supplement: S1 File — (DOCX) [file pone.0255904.s001.docx]
